# Identification of a Solute Carrier Family-Based Signature for Predicting Overall Survival in Osteosarcoma

**DOI:** 10.3389/fgene.2022.849789

**Published:** 2022-04-19

**Authors:** Di Zheng, Zhun Wei, Weichun Guo

**Affiliations:** Department of Orthopedics, Renmin Hospital of Wuhan University, Wuhan, China

**Keywords:** solute carrier, osteosarcoma, prognosis, signature, immune microenvironment

## Abstract

Given the important role of SLC family in essential physiological processes including nutrient uptake, ion transport, and waste removal, and that their dysregulation was found in distinct forms of cancer, here we identified a novel gene signature of SLC family for patient risk stratification in osteosarcoma. Gene expression data and relevant clinical materials of osteosarcoma samples were retrieved from The Cancer Genome Atlas (TCGA) database. Prognosis-related SLC genes were identified by performing univariate Cox regression analysis and were utilized to construct a four-SLC gene signature in osteosarcoma. It allowed patients to be classified into high- and low-risk groups, and Kaplan-Meier survival analysis in the training, testing, entire, and external GSE21257 cohorts suggested that the overall survival of patients in high-risk group was consistently worse than that in low-risk group, suggesting the promising accuracy and generalizability of the SLC-based signature in predicting the prognosis of patients with osteosarcoma. Moreover, univariate and multivariate Cox regression analyses indicated that the derived risk score was the only independent prognostic factor for osteosarcoma patients in TCGA and GSE21257 cohorts. Besides, a prognostic nomogram comprising the derived risk score and clinical features including gender and age was developed for clinical decision-making. Functional enrichment analyses of the differentially expressed genes between high- and low-risk group revealed that immune-related biological processes and pathways were significantly enriched. Estimation of tumor immune microenvironment using ESTIMATE algorithm revealed that patients with lower risk score had higher stromal, immune, and ESTIMATE score, and lower tumor purity. ssGSEA analyses indicated that the scores of various immune subpopulations including CD8+ T cells, DCs, and TIL were lower in high-risk group than these in low-risk group in both cohorts. As for the related immune functions, the scores of APC co-inhibition, CCR, check-point, T cell co-stimulation, and Type II IFN response were lower in high-risk group than these in low-risk group in both cohorts. In all, we identified a novel prognostic signature based on four SLC family genes that accurately predicted overall survival in osteosarcoma patients. Furthermore, the signature is linked to differences in immunological status and immune cell infiltrations in the tumor microenvironment.

## Introduction

Osteosarcoma, originating from the mesenchymal stem cells, is the most widespread primary malignancy of bone (Saraf et al., 2018). Osteosarcoma mainly affects teenagers and young adults and usually occurs in the distal femur and proximal tibia metaphyses (Lin et al., 2017). Despite the fact that osteosarcoma is an uncommon illness with an incidence of 4.4 per million yearly, it is particularly malignant that tends to metastasis at early stage (Dean et al., 2018; Fan et al., 2020). Currently, the conventional treatment for osteosarcoma includes surgical resection and neoadjuvant chemotherapy, which has boosted the 5-years survival rate for patients with localized osteosarcoma to 60%–70% (Gill and Gorlick, 2021; Meltzer and Helman, 2021). Unfortunately, the prognosis for patients with metastatic, recurrent, or unresectable osteosarcoma is exceedingly poor, with a 5-years survival rate of fewer than 20% (Nørregaard et al., 2021). In addition, the clinical outcome of patients with osteosarcoma varies even they have the same clinicopathological conditions and are treated under standard management due to their genetic heterogeneity (Gianferante et al., 2017; Rickel et al., 2017; Czarnecka et al., 2020). To yet, it has been impossible to properly predict the clinical fate of patients with osteosarcoma. As a result, there is an urgent need to uncover reliable predictive biomarkers to aid in patient risk classification and the development of individualized therapy and care strategies.

Solute carrier transporters (SLC) are a vast superfamily of transmembrane proteins with about 400 members (Pizzagalli et al., 2021). Though SLC proteins have received little attention in recent years, they are involved in the controlled transport of a variety of substrates as well as crucial physiological activities including nutrition intake, ion transport, and waste elimination (César-Razquin et al., 2015; Lin et al., 2015). SLC protein alterations have been observed in a variety of illnesses, including cancer, indicating that they might be used as therapeutic targets (Schumann et al., 2020; Sutherland et al., 2020). Indeed, certain SLC proteins have a role in the cellular absorption of cancer medicines such as chemotherapeutics and targeted therapies, and various therapeutic methods targeting SLC family members have been found and are being tested in cancer clinical trials (César-Razquin et al., 2015; Nyquist et al., 2017; Rives et al., 2017; Panda et al., 2020). Dysregulated SLCs have been linked to increased cell proliferation and invasion in osteosarcoma, and they may be diagnostic or prognostic indications in individuals with osteosarcoma (Zhu et al., 2017; Chen and Wu, 2018). Nonetheless, the clinical significance and roles of SLCs proteins in osteosarcoma need to be investigated further.

Here, we conducted a thorough investigation of SLC genes in osteosarcoma using data from public sources including The Cancer Genome Atlas (TCGA) and Gene Expression Omnibus (GEO). We discovered prognosis-related SLC genes in osteosarcoma and utilized them to develop a unique four-gene signature with predictive value in osteosarcoma. Following that, the accuracy and generalizability of the SLC-based signature in predicting the prognosis of osteosarcoma patients were validated in internal and external cohorts. We also created a nomogram for clinical decision-making. Finally, we explored the correlation between SLC-based signatures and tumor immune microenvironment and immune cell infiltration.

## Materials and Methods

### Data Collection

Transcriptome profiling data in fragment per kilobase method (FPKM) format and corresponding clinical information of osteosarcoma patients were downloaded from The Cancer Genome Atlas (https://portal.gdc.cancer.gov/). For external validation, the GSE21257 dataset (Buddingh et al., 2011) comprising the mRNA expression profile and clinical data was downloaded from the Gene Expression Omnibus database (https://www.ncbi.nlm.nih.gov/geo/). Patients whose follow-up time or survival status were missing were eliminated from the future analyses, and a total of 139 cases with complete information including 86 cases from TCGA cohort and 53 cases from GSE21257 cohort was enrolled in our study. The *sva* package in R was employed to eliminate the batch effects from non-biological technical biases between different datasets (Xu et al., 2021c).

### Identification of Prognosis-Related SLC Genes

The list of SLC family genes was compiled using information from the Human Gene Database (https://www.genecards.org/) and previous research (César-Razquin et al., 2015). These SLC genes were subjected to univariate Cox regression analysis after being assigned in a uniform nomenclature in both the TCGA osteosarcoma dataset and the GSE21257 dataset. In univariate Cox regression analysis, SLC genes with *p*-value less than 0.05 were identified as prognosis-related SLC genes.

### Construction and Validation of the SLC Gene-Based Signature

To create an SLC gene-based signature in osteosarcoma, we randomly split the TCGA (entire) cohort into a training and a testing cohort at a ratio of around 1:1. In the training cohort, prognosis-related SLC genes were submitted to LASSO regression analysis to exclude overfitting genes [By employing the *glmnet* and *survival* packages (Tibshirani, 1997; Engebretsen and Bohlin, 2019) in R]. Then, using multivariate Cox regression analysis, the SLC genes that were most significantly associated with prognosis were identified and the corresponding regression coefficients were obtained. The risk score of an osteosarcoma patient was determined using the following formula: $\mathrm{risk}\;\mathrm{score}=\;\sum_{\text{i}}^{\text{n}}{\text{Exp}}_{\text{i}}{\text{Coe}}_{\text{I}}$ , where Exp is the expression of SLCs and Coe is the regression coefficient. Following that, the risk scores of patients in the training, testing, entire, and external GSE21257 cohorts were determined, allowing patients to be further divided into high- and low-risk groups based on the median risk score in the training cohort. Using the *survival* and *survminer* packages (Wang et al., 2020b) in R, Kaplan-Meier survival analysis was performed to assess overall survival between high- and low-risk groups. To examine the specificity and sensitivity of the prognostic signature, time-dependent receiver operating characteristic (ROC) curves were calculated using the *survivalROC* package (Heagerty et al., 2000; Huang et al., 2020) in R.

### Construction and Validation of a Nomogram in Osteosarcoma

The *rms* package (Zhang et al., 2020b; Xu et al., 2021a) in R was used to create a predictive nomogram that included clinicopathological characteristics such as gender and age, as well as risk score based on the four-SLC gene signature. To examine the prediction efficacy of the nomogram in the TCGA and GSE21257 cohorts, calibration curves at various time periods including 1, 3, and 5 years were drawn.

### Identification of Risk-Related Differentially Expressed Genes and Function Analysis

Based on the risk score of each sample, the expression profiles of the TCGA entire cohort were split into high- and low-risk groups. Then, using the *edgeR* package (Robinson et al., 2010) in R and the criterion of *p*-value < 0.05 and | log2FC |> 0.5, differentially expressed genes (DEGs) between high- and low-risk groups were identified. Following that, DEGs were analyzed for Gene Ontology (GO) and Kyoto Encyclopedia of Genes and Genomes (KEGG) pathway enrichment using *clusterProfiler* package (Yu et al., 2012) in R. Furthermore, gene set enrichment analysis (GSEA) was used to uncover pathways that were significantly enriched in high- or low-risk groups using GSEA software (version 4.0.2, https://www.gsea-msigdb.org/gsea/index.jsp). We analyzed the gene sets of “c2.cp.kegg.v7.5.symbols.gmt [Curated]” from the Molecular Signatures Database through GSEA (Subramanian et al., 2005).

### Estimating Tumor Immune Microenvironment and Immune Cell Infiltration

The ESTIMATE algorithm (Yoshihara et al., 2013) was used to calculate the stromal score, immune score, ESTIMATE score, and further predicting tumor purity in each osteosarcoma sample. Kaplan-Meier survival analysis was used to compare the overall survival of patients with different stromal, immune, and ESTIMATE scores, as well as tumor purities. Using the CIBERSORT algorithm (Newman et al., 2015), the proportion of 22 immune cell subtypes infiltrated in each sample was computed and compared between high- and low-risk groups. Furthermore, single-sample gene set enrichment analysis (ssGSEA) was used to assess the scores of distinct infiltrating immune subpopulations and related-immune functions in each TCGA and GSE21257 osteosarcoma sample (Hänzelmann et al., 2013; Zuo et al., 2020).

### Statistical Analysis

The R software (version 4.1.0) was used for all of the statistical analyses. The log-rank test was used in Kaplan-Meier survival analysis to assess the difference in overall survival between high- and low-risk groups. To compare gene expression or scores between high- and low-risk groups, the two-tailed Student’s t-test or Wilcoxon test were used. Statistical significance was defined as a *p*-value less than 0.05.

## Results

### Identification of Prognosis-Related Genes in SLC Family

**FIGURE 1:**
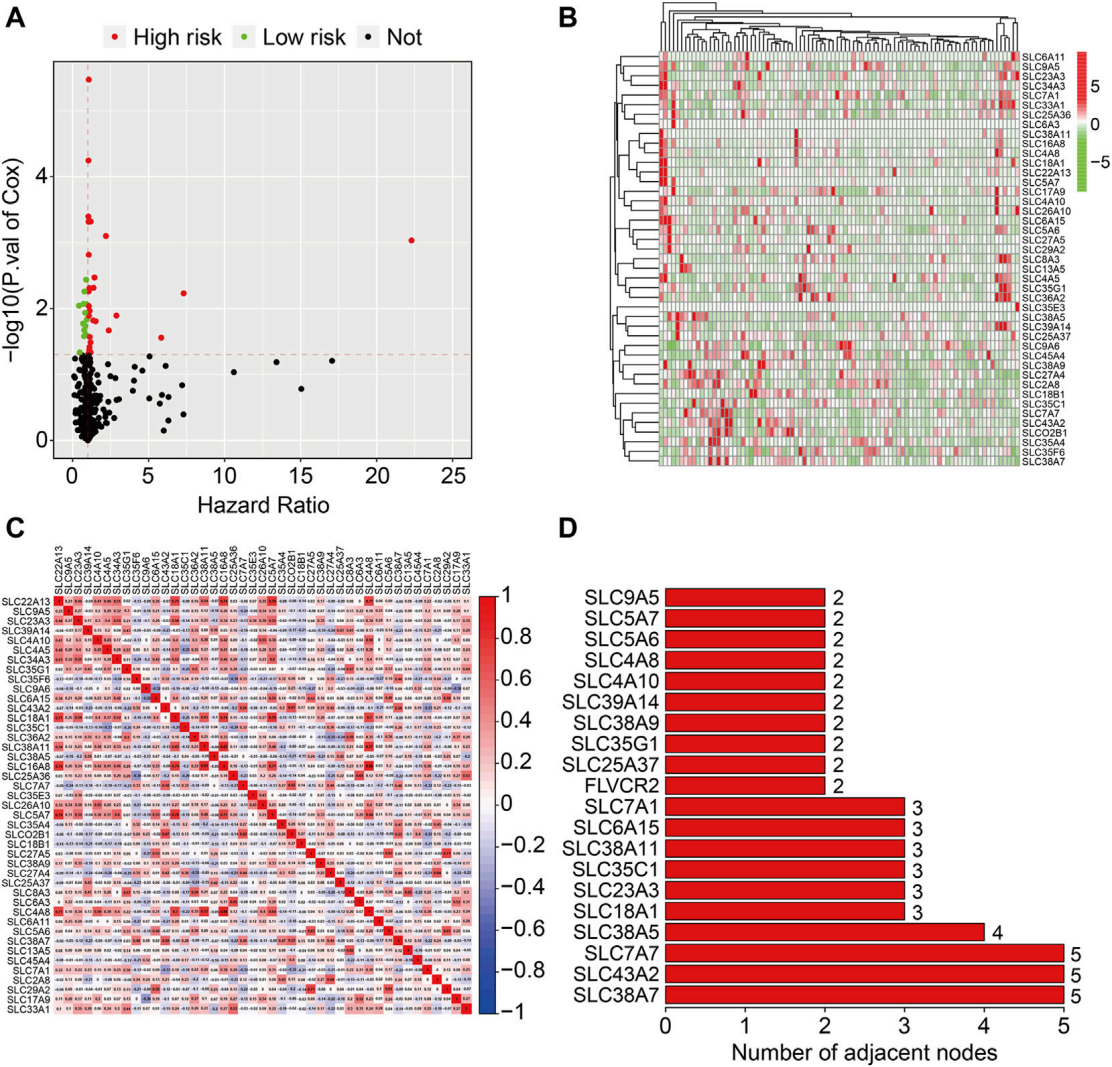
Identification of prognosis-related SLC family genes in osteosarcoma. **(A)** Volcano plot presented prognosis-related genes of SLC family. **(B)** Heatmap depicted the expression patterns of prognosis-related SLC family genes in dataset from the TCGA database. **(C)** The correlation heatmap of the prognosis-related SLC family genes. **(D)** The bar chart displayed the number of adjacent nodes in PPI network.

We used univariate Cox regression analysis on datasets from the TCGA database to discover prognosis-related genes of the SLC family in osteosarcoma. Forty four SLC genes with *p*-values less than 0.05 in univariate Cox regression analysis were identified as SLC family prognosis-related genes (Figure 1A) and the detailed information of these SLC genes were shown in Table 1. In osteosarcoma, 31 of the 44 SLC genes were risk factors (HR > 1), whereas 13 were protective factors (HR < 1). Figure 1B depicts the expression profile of SLC family prognosis-related genes. The correlation of these SLC family prognosis-related genes is shown in Figure 1C. Furthermore, using the STRING online tool, these prognosis-related SLC genes were used to build a protein-protein interaction (PPI) network. The top three ranking genes in the PPI network were *SLC38A7*, *SLC7A7*, and *SLC43A2* (Figure 1D).

**TABLE 1:** The detailed information of prognosis-related SLC genes in the univariate Cox regression analysis.

Gene	HR	HR.95L	HR.95H	*p* value
SLC8A3	1.062230	1.035522	1.089626	3.38E-06
SLC39A14	1.042375	1.021522	1.063654	5.69E-05
SLC38A5	1.033604	1.014851	1.052705	0.000403
SLC29A2	1.188300	1.078643	1.309104	0.000479
SLC17A9	1.047965	1.020749	1.075907	0.000484
SLC27A5	2.190054	1.385174	3.462624	0.000797
SLC34A3	22.29727	3.549565	140.0645	0.000929
SLC22A13	85.11842	5.991972	1209.142	0.001029
SLC16A8	1.062055	1.023227	1.102357	0.001533
SLC4A8	1.435703	1.127287	1.828499	0.003379
SLC27A4	0.887577	0.818997	0.961899	0.003652
SLC35G1	1.375200	1.101799	1.716442	0.004845
SLC5A6	1.098855	1.029022	1.173427	0.004894
SLC13A5	1.063661	1.018358	1.110980	0.005450
SLC7A7	0.773336	0.644981	0.927234	0.005506
SLC23A3	7.301993	1.775131	30.03671	0.005864
SLC38A7	0.712624	0.553673	0.917206	0.008510
SLC35A4	0.916776	0.859262	0.978140	0.008574
SLC45A4	0.417194	0.216412	0.804258	0.009043
SLC36A2	1.069440	1.016775	1.124832	0.009170
SLC6A15	1.114349	1.025298	1.211133	0.010838
SLCO2B1	0.820543	0.703751	0.956718	0.011577
SLC26A10	2.880016	1.252662	6.621495	0.012763
SLC7A1	1.073544	1.015181	1.135263	0.012838
SLC4A5	82.81834	2.463924	2783.721	0.013786
SLC35F6	0.930447	0.878111	0.985902	0.014661
SLC6A11	1.356341	1.060603	1.734542	0.015147
SLC9A5	1.514783	1.082144	2.120391	0.015520
SLC5A7	205.4600	2.685417	15719.64	0.016113
SLC43A2	0.685212	0.502525	0.934313	0.016875
SLC18B1	0.817505	0.691504	0.966465	0.018306
SLC2A8	0.762801	0.605886	0.960354	0.021210
SLC4A10	2.376891	1.136484	4.971131	0.021459
SLC35C1	0.864104	0.760783	0.981456	0.024572
SLC9A6	0.752133	0.585089	0.966868	0.026222
SLC38A11	1.125721	1.013678	1.250149	0.026833
SLC18A1	5.828358	1.214499	27.97018	0.027608
SLC6A3	1.179392	1.013838	1.371980	0.032513
SLC49A2	1.109039	1.005563	1.223162	0.038361
SLC25A36	1.093407	1.002976	1.191992	0.042618
SLC25A37	1.123292	1.002326	1.258856	0.045508
SLC33A1	1.183141	1.002911	1.395759	0.046104
SLC38A9	0.467541	0.221452	0.987100	0.046150
SLC35E3	1.037921	1.000259	1.077001	0.048416

### Construction of a Four-Gene Signature of the SLC Gene Family in Training Cohort

**FIGURE 2:**
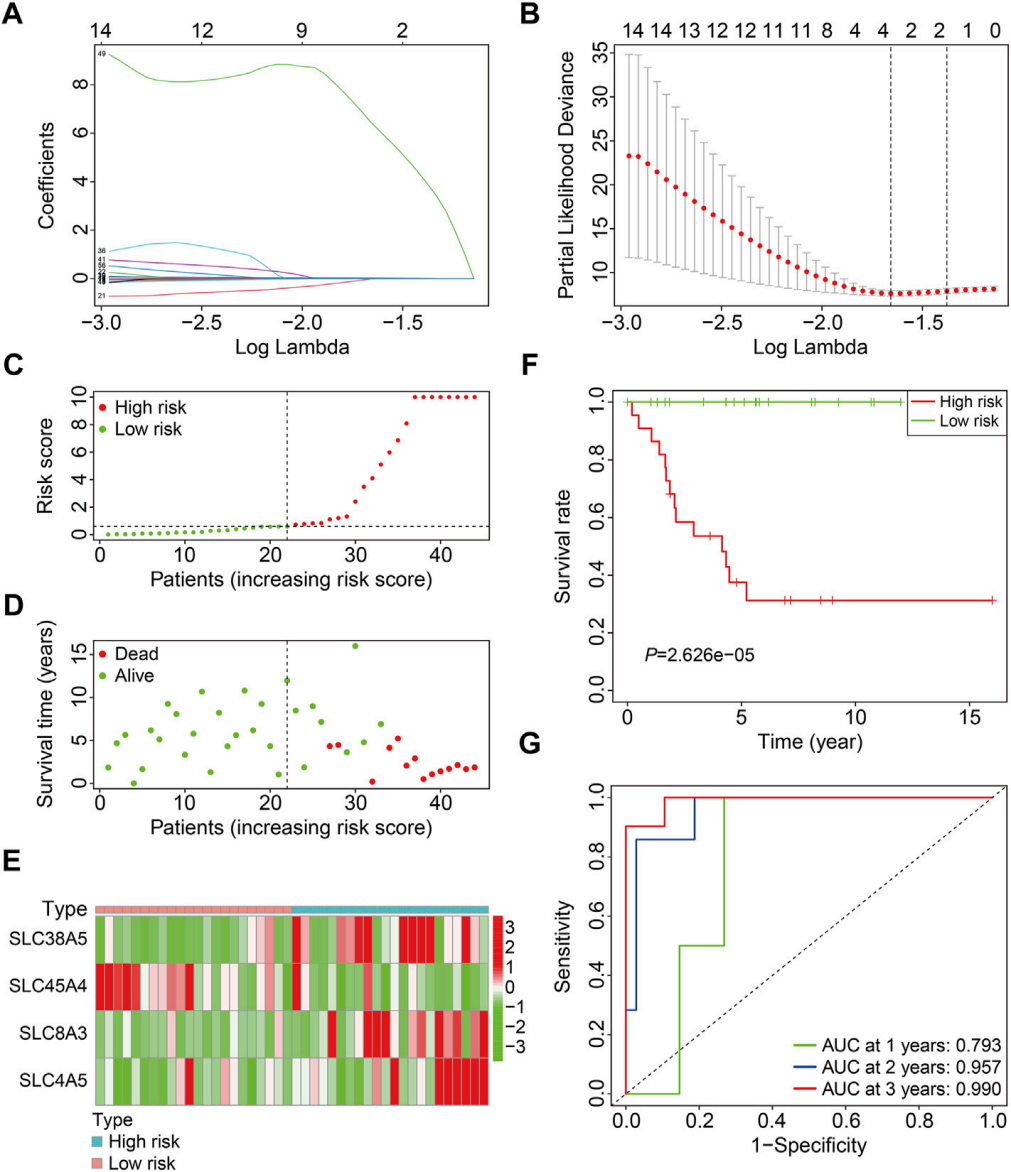
Construction of a SLC family gene-based signature in the training cohort. **(A)** The regression coefficients of the SLC genes for the different values of the penalty parameter (lambda) in the Lasso regression analysis. **(B)** The 10-fold cross-validation method is used to select the penalty parameter (lambda). The dashed line represents two lambda that produced automatically in the Lasso analysis, one that minimizes the binomial deviance and the other one representing largest λ that is still within 1 standard error of the minimum binomial deviance. **(C)** Distribution of patients’ risk score in training cohort. The horizontal dashed line represents the median risk score. The vertical dashed line is the dividing line that separates patients into high- and low-risk groups. **(D)** Distribution of survival status and survival time of patients in training cohort. **(E)** The expression profile of the four SLC genes in training cohort. **(F)** Kaplan-Meier survival curves. **(G)** Time-dependent receiver operating characteristic curve.

In the training cohort (*n* = 44), prognosis-related SLC genes were submitted to LASSO regression analysis, followed by multivariate Cox regression analysis (Figures 2A,B), resulting in an SLC-based prognostic signature containing four SLC family genes, including *SLC38A5*, *SLC45A4*, *SLC8A3*, and *SLC4A5*. The risk score for each instance was determined using a linear combination of these coefficients and the expression levels of the four SLC genes, as follows: risk score = *SLC38A5* × (0.111) + *SLC45A4* × (−1.958) + *SLC8A3* × (0.086) + *SLC4A5* × (18.310). Following that, the risk scores of patients in the training cohort were determined, allowing patients to be separated into high- and low-risk groups based on the median risk score (Figure 2C). Figure 2D depicts the survival status and survival time of patients in high- and low-risk groups, and the scatter plot revealed that patients in high-risk groups had a higher death rate. The expression patterns of the four SLC genes in the training cohort’s high- and low-risk groups are shown in Figure 2E. Furthermore, we used Kaplan-Meier survival analysis to evaluate overall survival between high- and low-risk groups, and the findings showed that patients in the high-risk group had a considerably poorer prognosis than those in the low-risk group (Figure 2F). Furthermore, time-dependent ROC curve analysis revealed that the AUC values for 1-, 2-, and 3-years overall survival were 0.793, 0.957, and 0.990 (Figure 2G), indicating that the SLC-based prognostic signature possessed promising accuracy and specificity for overall survival prediction in osteosarcoma patients.

### Validation of Four-Gene Signature of the SLC Gene Family in Internal Cohorts

**FIGURE 3:**
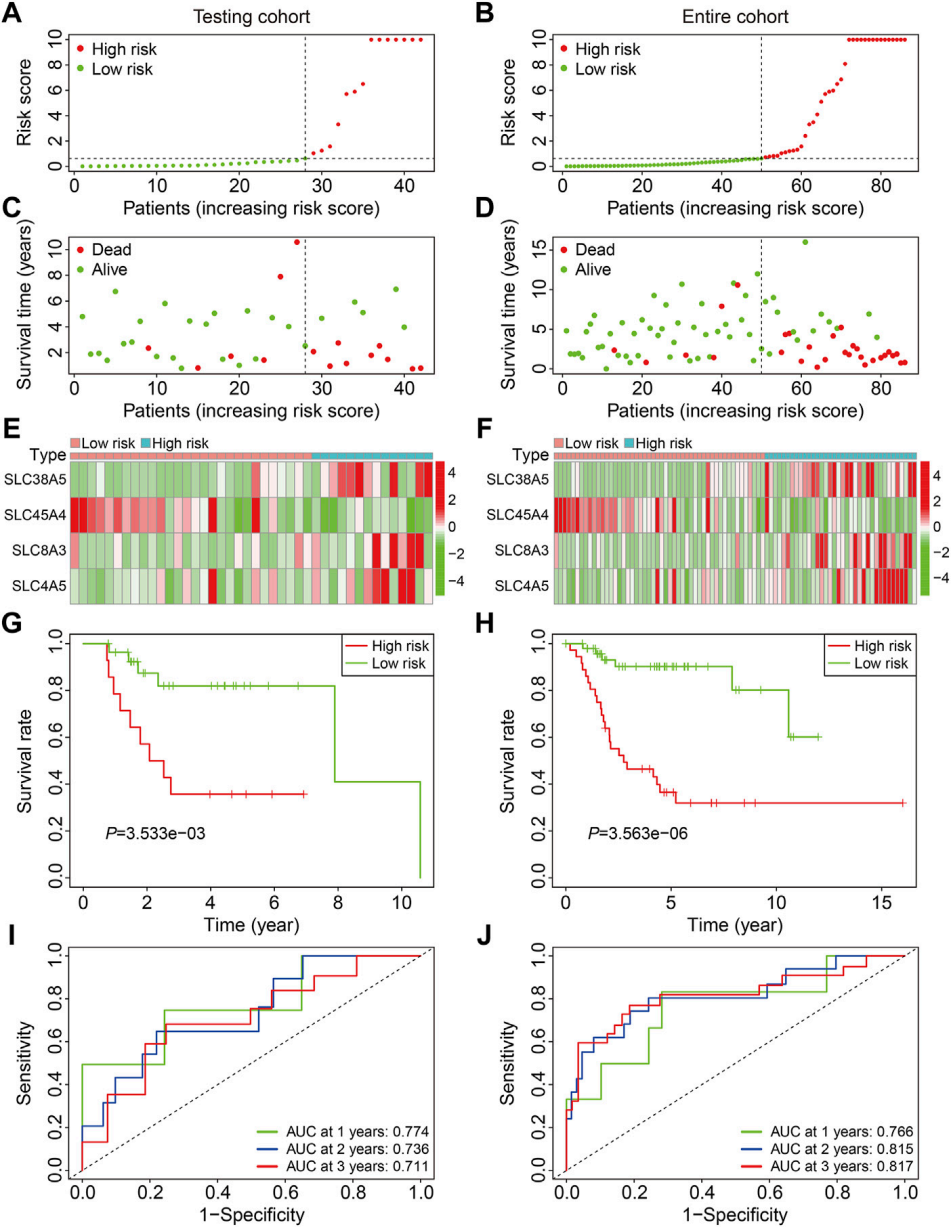
Validation of the SLC gene signature in internal cohorts. **(A,B)** Distribution of patients’ risk score in testing and entire cohorts. The horizontal dashed line represents the median risk score in training cohort. The vertical dashed line is the dividing line that separates patients into high- and low-risk groups. **(C,D)** Distribution of survival status and survival time of patients in testing and entire cohorts. **(E,F)** The expression profile of the four SLC genes in testing and entire cohorts. **(G,H)** Kaplan-Meier survival curves in testing and entire cohorts. **(I,J)** Time-dependent receiver operating characteristic curve analysis for the signature in testing and entire cohorts.

We first confirmed the SLC-based prognostic signature’s accuracy in internal cohorts, including the testing cohort (*n* = 42) and the entire cohort (*n* = 86). The risk scores of patients were determined using the aforementioned algorithm, and patients were divided into high- and low-risk groups based on the median risk score in the training cohort. Figures 3A,B depict the risk score distribution of patients in testing and entire cohorts. The survival status and survival time of patients in testing and entire cohorts are shown in Figures 3C,D, and as predicted, patients in the high-risk group were more likely to die. Figures 3E,F show the expression patterns of the four SLC genes in patients with varying risk scores from testing and entire cohorts. Kaplan-Meier survival analysis indicated a substantial difference in overall survival between the high-risk and low-risk groups in testing and entire cohorts (Figures 3G,H). The AUC values of the ROC curves in the test cohort were 0.774 at 1 year, 0.736 at 2 years, and 0.711 at 3 years (Figure 3I), and 0.766 at 1 year, 0.815 at 2 years, and 0.817 at 3 years in the entire cohort (Figure 3J).

### Validation of Four-Gene Signature of the SLC Gene Family in External Cohort

**FIGURE 4:**
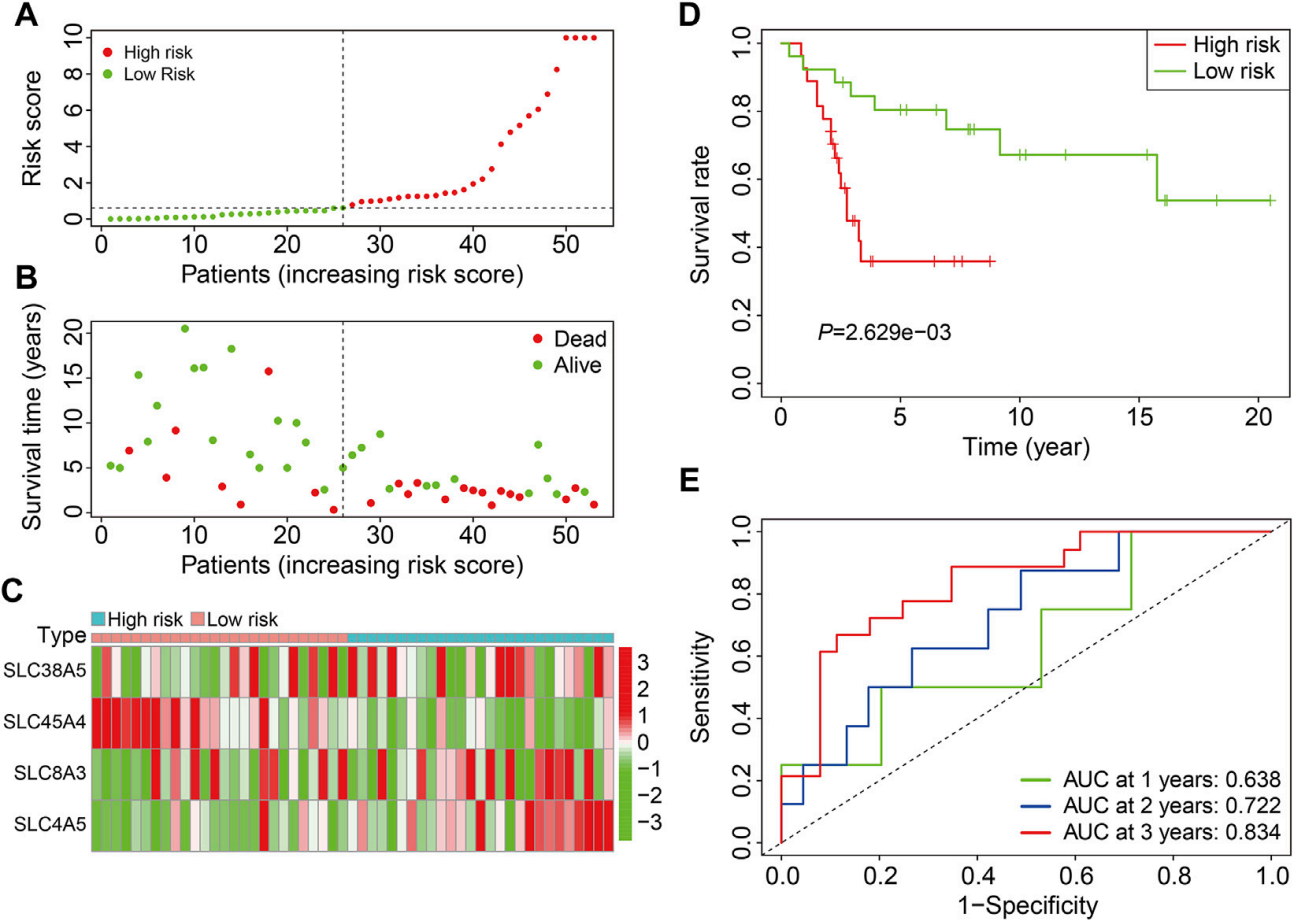
Validation of the SLC gene signature in GSE21257 cohort. **(A)** Distribution of patients’ risk score in GSE21257 cohort. **(B)** Distribution of survival status and survival time of patients in GSE21257 cohort. **(C)** The expression profile of the four SLC genes in GSE21257 cohort. **(D)** Kaplan-Meier survival curves. **(E)** Time-dependent receiver operating characteristic curve analysis for the signature in external GSE21257 cohort.

The GSE21257 cohort (*n* = 53) was used for external validation to further validate the accuracy and generalizability of the prognostic signature based on the four SLCs. As previously stated, patients in the GSE21257 cohort were divided into high- and low-risk groups (Figure 4A). The survival status and survival time of patients in the external cohort are depicted in Figure 4B, and the results demonstrate that patients with a higher risk score were more likely to die and survived for a shorter period. Figure 4C depicts the expression patterns of the four SLC genes in high- and low-risk groups. Kaplan-Meier survival analysis suggested the overall survival of patients in the low-risk group was higher than that of patients in the high-risk group (Figure 4D). The AUC values of the ROC curves were 0.638 at 1 year, 0.722 at 2 years, and 0.834 at 3 years, demonstrating that the SLC gene family-based prognostic signature was accurate and specific in predicting survival outcomes in osteosarcoma patients (Figure 4E).

### Stratified Analysis of the Four-Gene Signature Based on Clinicopathological Features

**FIGURE 5:**
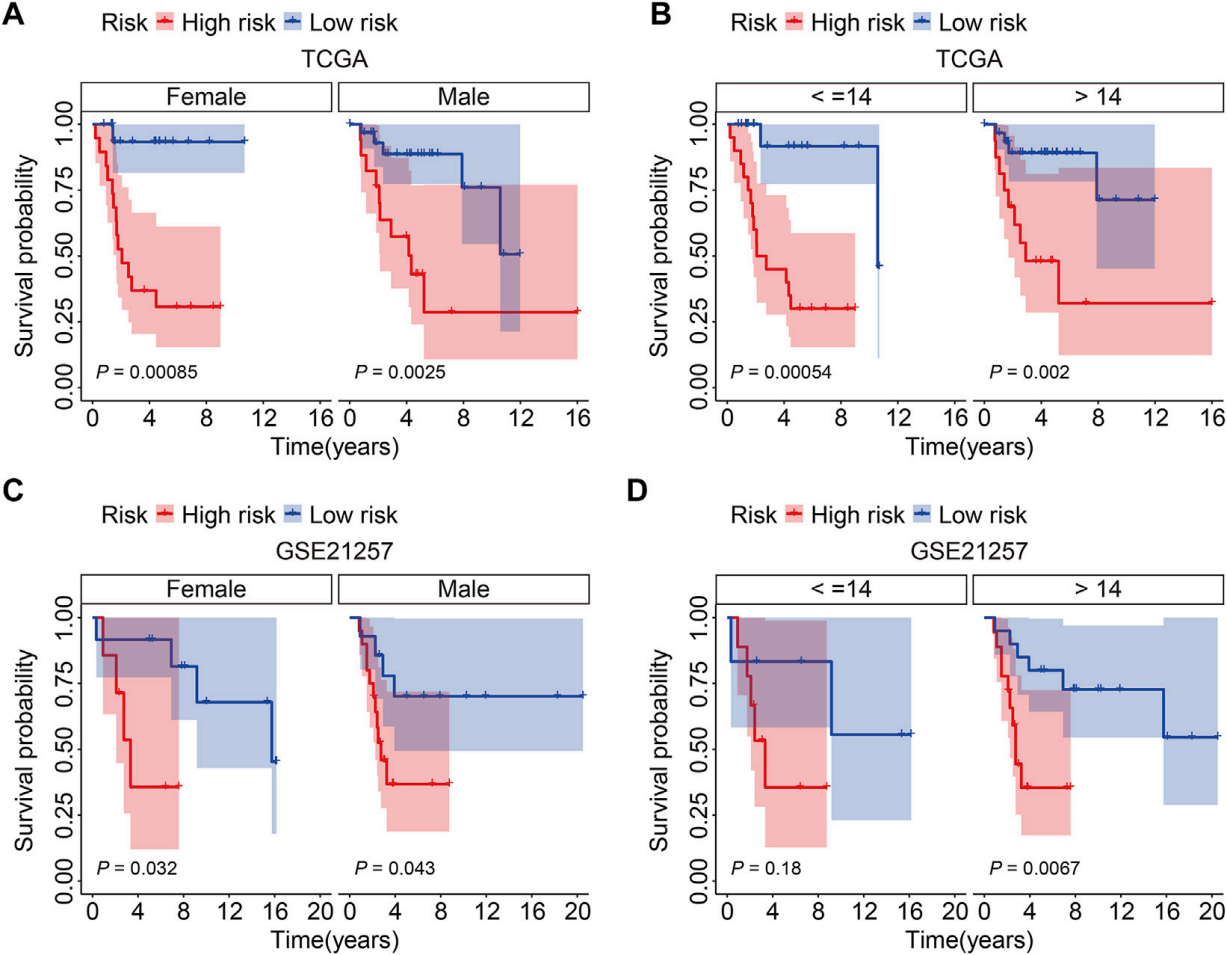
Kaplan-Meier survival analysis in subgroups classified by gender (female, male) or age (≤14, >14) in TCGA cohort **(A,B)** and GSE21257 cohort **(C,D)**.

Patients in the TCGA entire cohort and the external GSE21257 cohort were separated into varied subgroups based on clinicopathological factors such as gender (female, male) and age (≤14, >14) to further establish the prognostic value of the SLC family gene-based signature. The overall survival of patients in the high-risk and low-risk groups was compared in each grouping, and the findings indicated that patients in the low-risk group tended to live longer in all categories (Figures 5A–D).

### The Prognostic Independence of the Four Gene Signature and Construction of a Nomogram in Osteosarcoma

**FIGURE 6:**
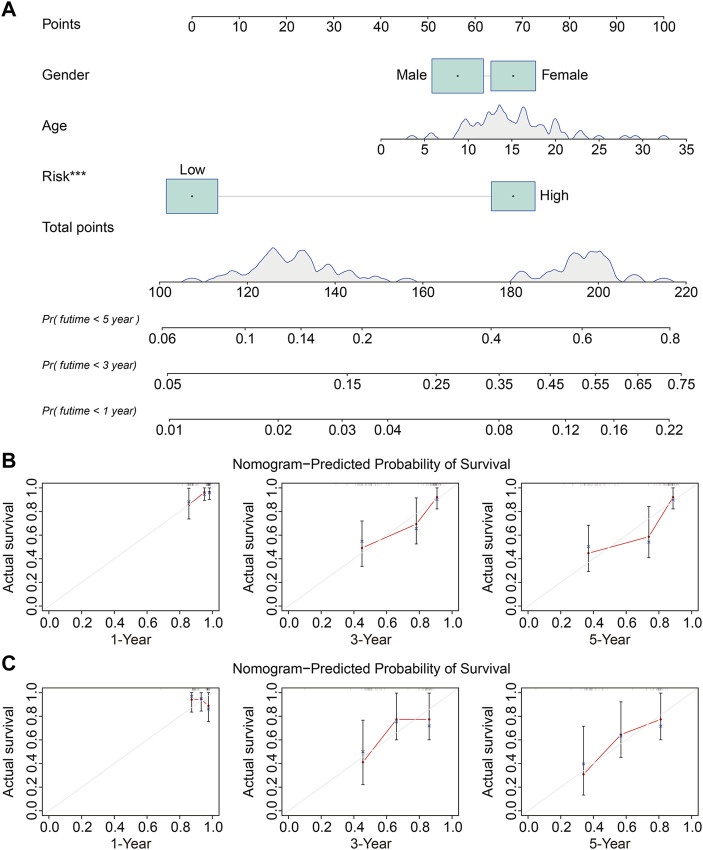
Construction and validation of a nomogram in TCGA osteosarcoma cohort and GSE21257 cohort. **(A)** The prognostic nomogram for predicting 1-, 3-, 5-years overall survival of osteosarcoma patients. **(B,C)** The calibration curves for 1-, 3-, 5-years overall survival prediction in TCGA entire cohort and external GSE21257 cohort. In a well-calibrated model, the predictions should fall on or close to the 45-degree diagonal line.

We used univariate and multivariate Cox regression analyses to determine the impact of clinicopathological variables and risk score on overall survival in the TCGA entire cohort and the external GSE21257 cohort of osteosarcoma patients. In both cohorts, the risk score based on the four gene signature was the sole independent predictive predictor, as shown in Tables 2, 3. To develop a quantitative technique for predicting the clinical prognosis of osteosarcoma patients, we created a prognostic nomogram that included gender, age, and risk score (Figure 6A). The calibration curves showed excellent agreement between the nomogram prediction and actual observation, suggesting the predicted values of 1-, 3-, and 5-years overall survival were closely matched with the actual values. It was consistent in both the TCGA entire cohort and the external GSE21257 cohort, demonstrating that the prognostic nomogram was trustworthy and accurate (Figures 6B,C).

**TABLE 2:** Univariable and multivariable analysis of the four-SLC prognostic signature and clinical factors in the TCGA cohort.

Variables	Univariable analysis				Multivariable analysis			
	HR	95% CI of HR		*p*	HR	95% CI of HR		*p*
		Lower	Upper			Lower	Upper	
Gender (female vs. male)	0.681	0.328	1.416	0.304	0.702	0.303	1.626	0.409
Age (≤14 vs. > 14)	0.652	0.313	1.355	0.252	0.650	0.278	1.518	0.319
Risk score	1.008	1.004	1.011	0.000	1.008	1.005	1.012	0.000

**TABLE 3:** Univariable and multivariable analysis of the four-SLC prognostic signature and clinical factors in the GSE21257 cohort.

Variables	Univariable analysis				Multivariable analysis			
	HR	95% CI of HR		*P*	HR	95% CI of HR		*p*
		Lower	Upper			Lower	Upper	
Gender (female vs. male)	1.403	0.588	3.348	0.445	2.071	0.787	5.451	0.140
Age (≤14 vs. > 14)	1.009	0.975	1.044	0.603	1.021	0.987	1.058	0.231
Risk score	1.049	1.017	1.083	0.003	1.065	1.027	1.104	0.001

### Functions and Pathways Correlated With the Four-SLC Gene Signature

**FIGURE 7:**
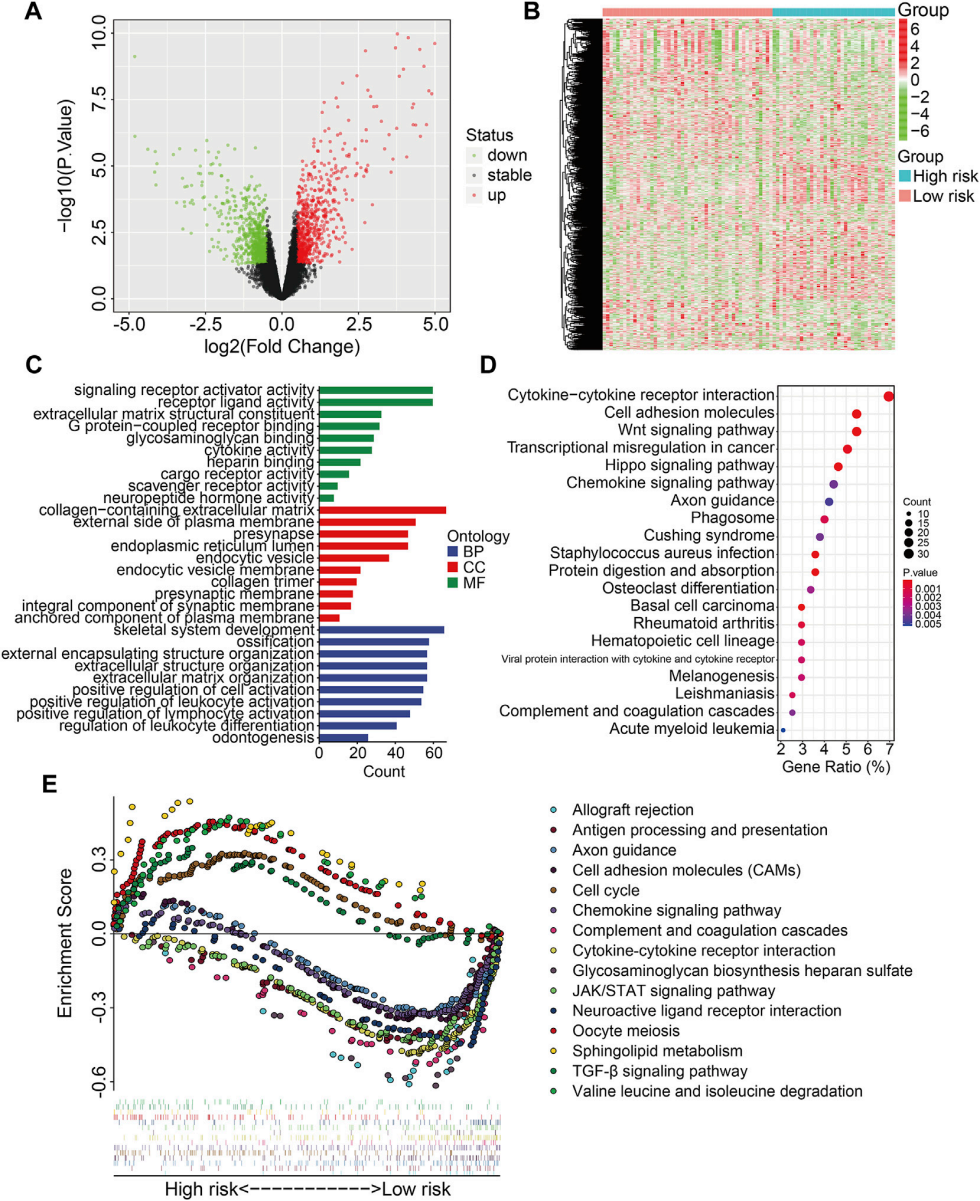
Identification of risk-related differentially expressed genes and functional enrichment analysis. **(A,B)** The volcano plot and heatmap exhibited differentially expressed genes between high- and low-risk groups. **(C)** GO enrichment analysis. **(D)** KEGG pathway enrichment analysis. **(E)** GSEA analysis between high- and low-risk groups. Each kind of color represents an enriched pathway.

We identified 1,432 differentially expressed genes (DEGs) between high- and low-risk groups in the TCGA entire cohort to investigate the potential biological processes and pathways associated with the signature. In the high-risk group, 724 of the 1,432 DEGs were upregulated, whereas 708 were downregulated (Figures 7A,B). DEGs were then submitted to GO and KEGG enrichment analyses. GO analysis revealed significant enrichment of biological processes including positive regulation of leukocyte activation, positive regulation of lymphocyte activation, and regulation of leukocyte differentiation. In terms of cellular components, collagen-containing extracellular matrix, external side of plasma membrane, and presynapse were the three most enriched terms. In terms of molecular function, DEGs were particularly enriched for signaling receptor activator activity, receptor ligand activity, and extracellular matrix structural constituent (Figure 7C). KEGG enrichment analysis revealed that DEGs mostly participated in cytokine-cytokine receptor interaction, cell adhesion molecules, the WNT signaling pathway, cancer transcriptional misregulation, and the Hippo signaling network (Figure 7D). Furthermore, gene set enrichment analysis was performed to compare the differences between the high-risk and low-risk groups, and the results revealed that immune-related pathways such as antigen processing and presentation, chemokine signaling pathway, complement and coagulation cascades, cytokine-cytokine receptor interaction, JAK/STAT signaling pathway, and TGF-β signaling pathway were significantly enriched (Figure 7E).

### Estimation of the Tumor Immune Microenvironment and Immune Cell Infiltration Using the Four- SLC Gene Signature

**FIGURE 8:**
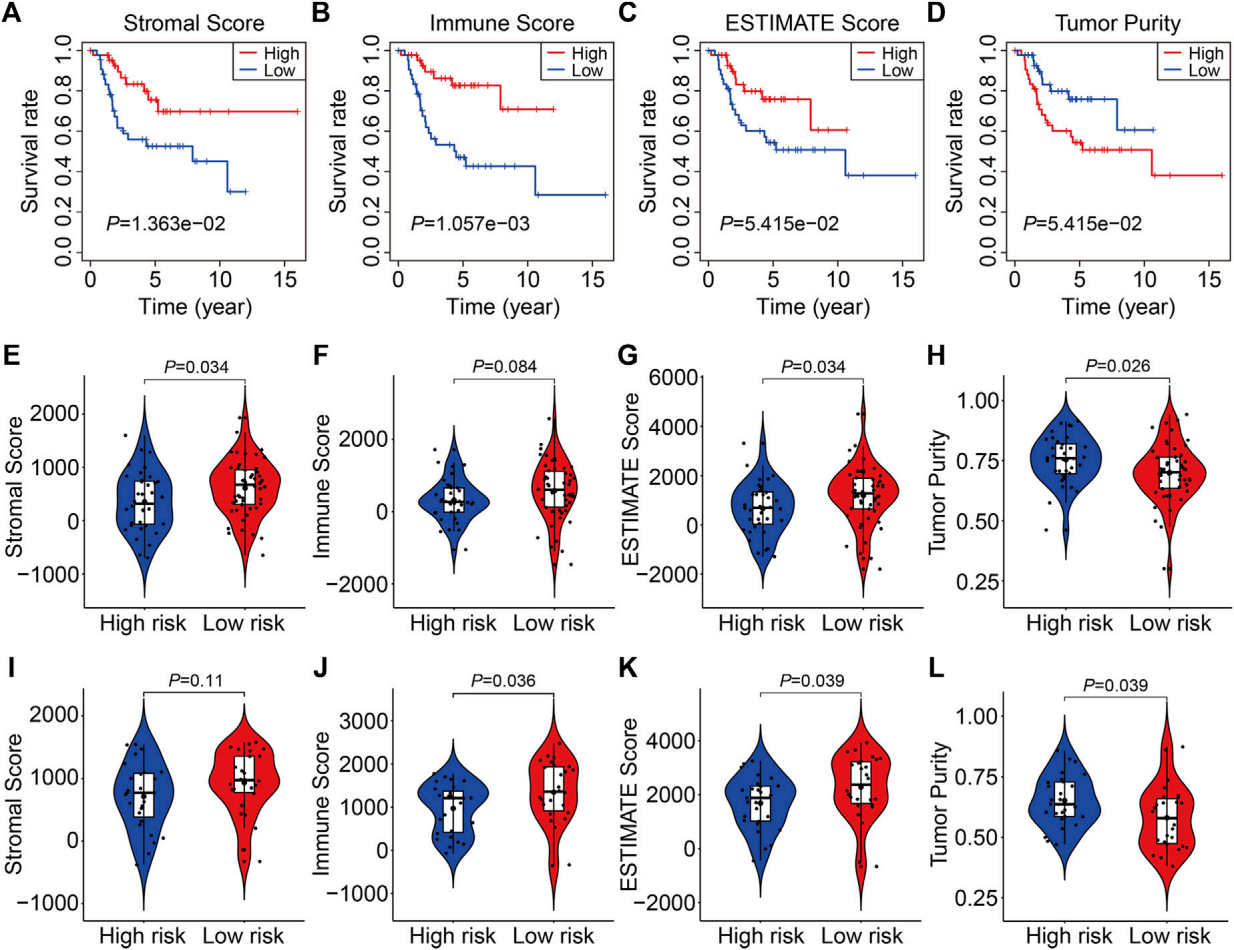
The four SLC gene signature was correlated with tumor immune microenvironment. **(A–D)** Kaplan-Meier survival analysis was utilized to compare the overall survival in patients with different stromal, immune, and ESTIMATE score, and tumor purity. **(E–H)** The stromal, immune, and ESTIMATE score, and tumor purity in high- and low-risk groups of the TCGA entire cohort. **(I–L)** The stromal, immune, and ESTIMATE score, and tumor purity in high- and low-risk groups of the GSE21257 cohort.

The stromal score, immune score, ESTIMATE score, and tumor purity in each TCGA and GSE21257 sample were quantified using the ESTIMATE algorithm to further investigate the indicative roles of SLC family-based signatures on immune microenvironment. As shown in Figures 8A–D, Kaplan-Meier survival analysis revealed that patients with higher stromal, immune, and ESTIMATE scores had better overall survival, and patients with higher tumor purity lived shorter lives than those with lower tumor purity, implying that the immune status of the immune microenvironment was an indicator of overall survival in patients with osteosarcoma. Following that, we compared the stromal score, immune score, ESTIMATE score, and tumor purity in high- and low-risk groups in TCGA and GSE21257 cohort. Consistently, patients with lower risk score had higher stromal scores, immune scores, ESTIMATE scores, and lower tumor purities (Figures 8E–L). Thus, our four-SLC gene signature was found to be related to the tumor immune microenvironment and may be utilized to predict the efficacy of immune treatment.

**FIGURE 9:**
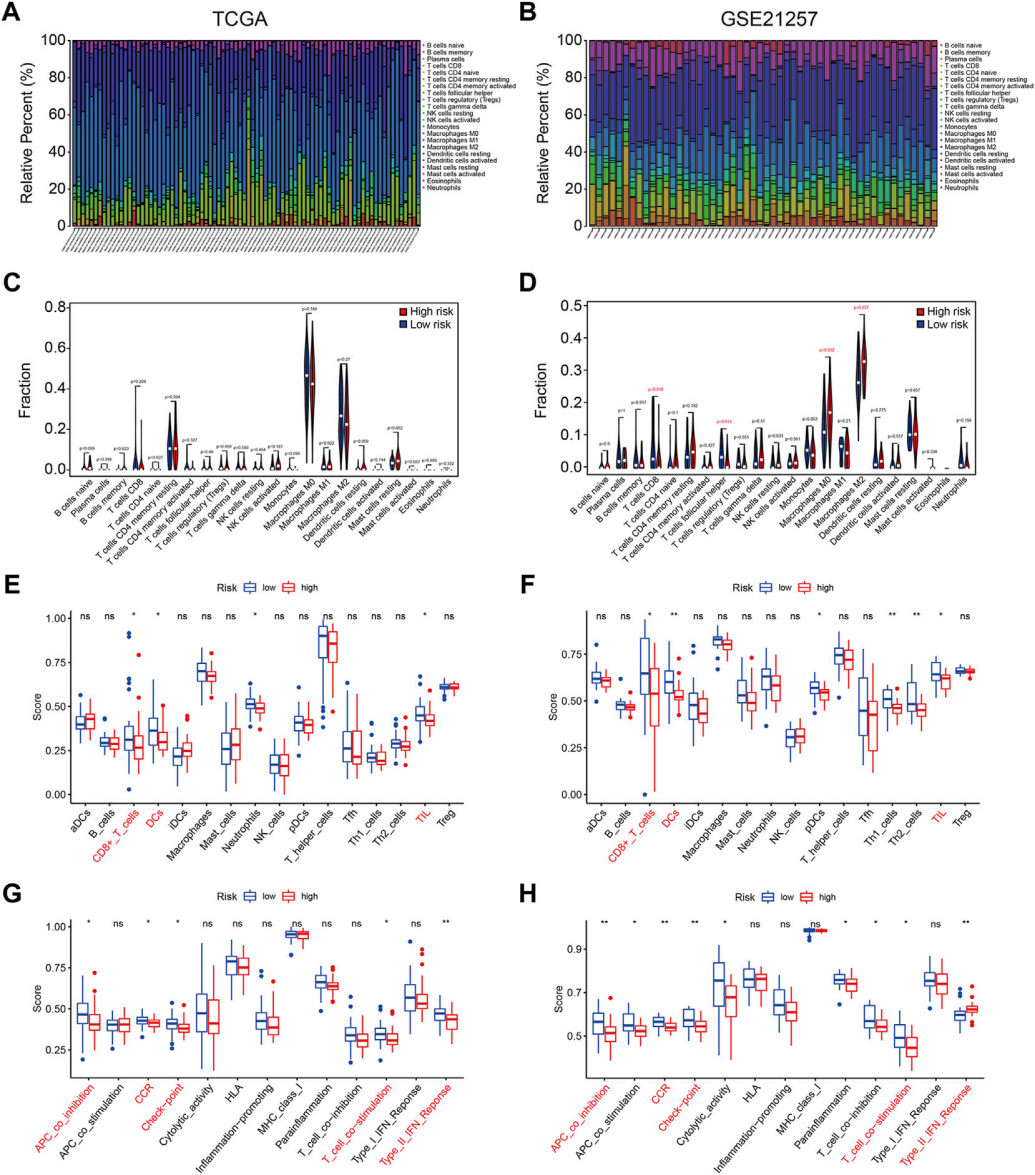
The four SLC gene signature was correlated with tumor immune cell infiltration. **(A,B)** Stacked bar chart exhibited the distribution of 22 immune cells in each osteosarcoma sample of the TCGA cohort and GSE21257 cohort. **(C,D)** Violin plots of immune cell infiltrates in high- and low-risk groups in TCGA cohort and GSE21257 cohort. **(E,F)** Box plots showed the scores of various immune subpopulations in high- and low-risk groups in TCGA cohort and GSE21257 cohort. **(G,H)** Box plots showed the scores of related-immune functions in high- and low-risk groups in TCGA cohort and GSE21257 cohort.

To further characterize the immune microenvironment, we used the CIBERSORT algorithm to assess the types of infiltrated immune cells in all TCGA and GSE21257 osteosarcoma samples. The fraction of 22 immune cell types assessed by the CIBERSORT algorithm revealed that M0 and M2 Macrophages and were the most prevalent immune cell types in the microenvironment, as illustrated in Figures 9A,B. In the TCGA cohort, there was no significant difference in the proportions of infiltrated immune cells between high- and low-risk groups (Figure 9C), whereas the proportions of infiltrating CD8 T cells, T follicular helper cells, M0 Macrophages and M2 Macrophages were statistically different between high- and low-risk groups in the GSE21257 cohort (Figure 9D). We also used ssGSEA to calculate the enrichment scores of various immune cell subpopulations and related immune functions. In both cohorts, the scores for several immune subpopulations such as CD8+ T cells, DCs, and TIL were lower in the high-risk group than in the low-risk group, as shown in Figures 9E,F. As for the related immune functions, the scores for APC co-inhibition, CCR, check-point, T cell co-stimulation, and Type II IFN response were lower in the high-risk group than in the low-risk group (Figures 9G,H). These findings revealed that the SLC-based signature might be associated with the immunological status of osteosarcoma patients.

### Expression and Kaplan-Meier Survival Analyses of the Four Genes of SLC Family

**FIGURE 10:**
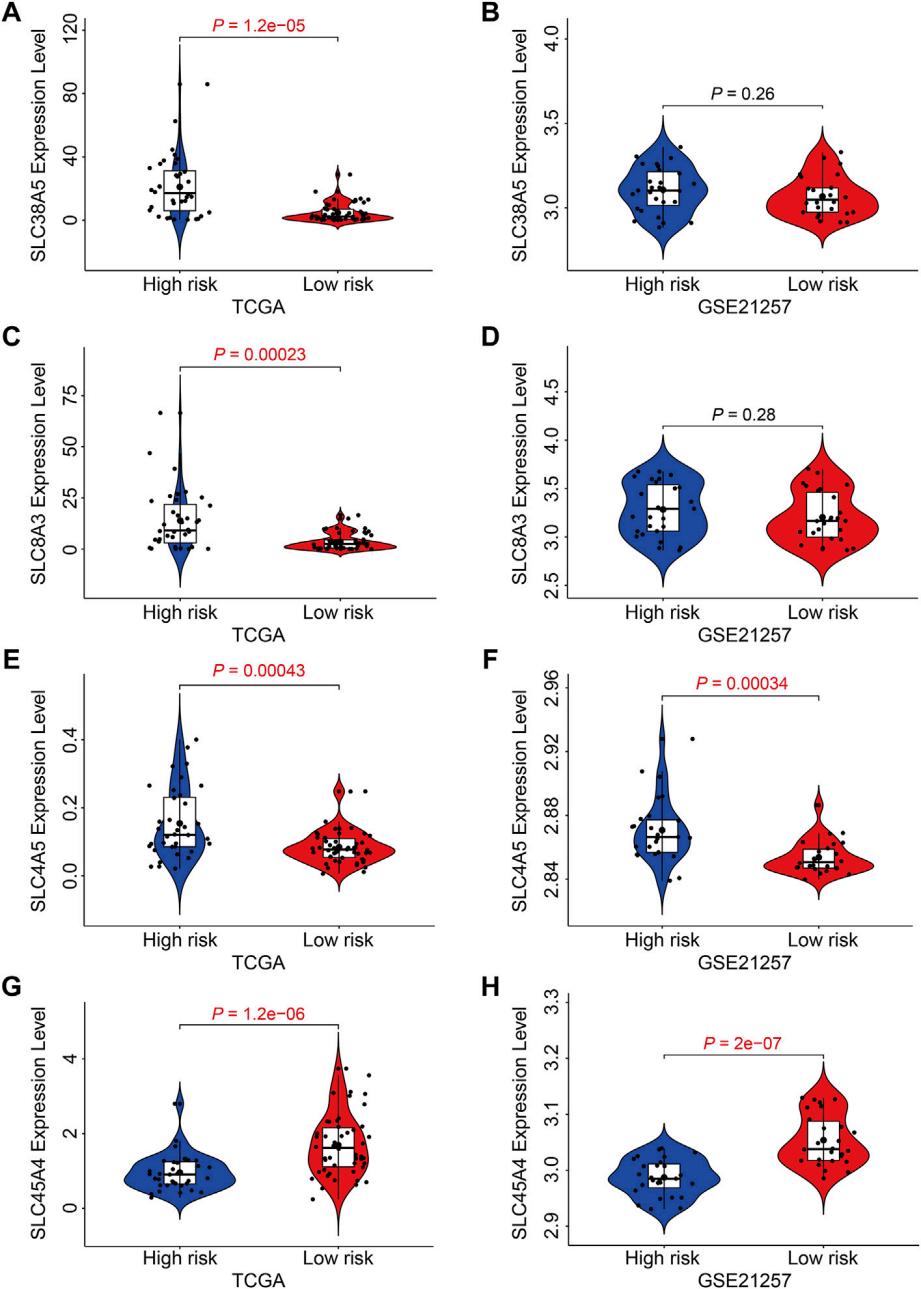
Expression analysis of the four SLC genes in high- and low-risk groups. **(A, B)** The expression of *SLC38A5* in TCGA and GSE21257 cohort. **(C,D)** The expression of *SLC8A3* in TCGA and GSE21257 cohort. **(E,F)** The expression of *SLC4A5* in TCGA and GSE21257 cohort. **(G,H)** The expression of *SLC45A4* in TCGA and GSE21257 cohort.

**FIGURE 11:**
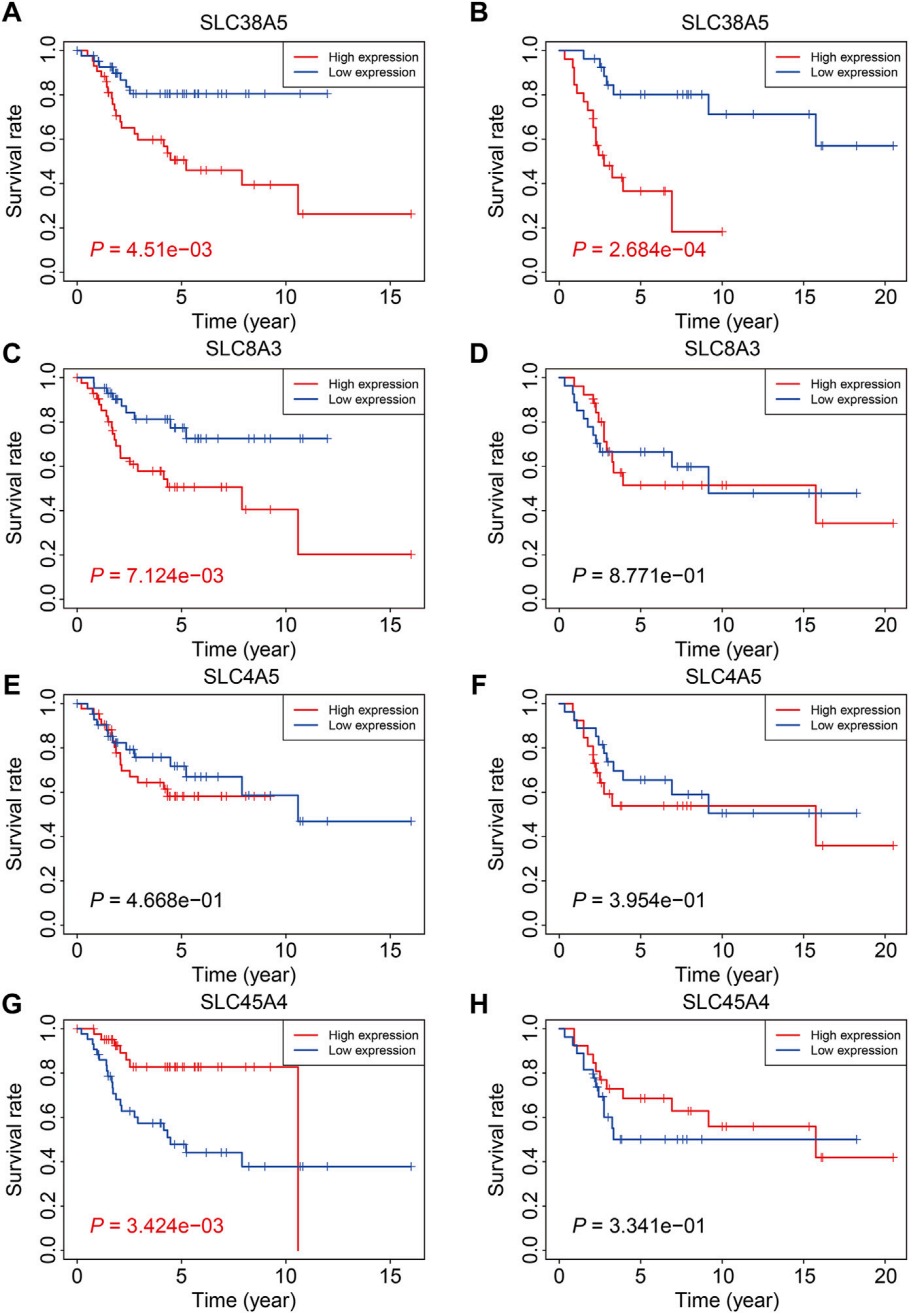
Kaplan-Meier survival analysis of the four SLC genes including *SLC38A5* **(A,B)**, *SLC8A3* **(C,D)**, *SLC4A5* **(E,F)**, and *SLC45A4* **(G,H)** in TCGA osteosarcoma cohort and GSE21257 cohort.

Finally, we examined the expression of the four SLC genes in the TCGA and GSE21257 datasets and performed a Kaplan-Meier survival analysis. As shown in Figures 10A–F, the expression levels of *SLC38A5*, *SLC8A3*, and *SLC4A5* were higher or trended to be higher in the high-risk group, but *SLC45A4* expression was lower in the high-risk group in both cohorts (Figures 10G,H). In the TCGA cohort, higher expression of *SLC38A5* and *SLC8A3*, and lower expression of *SLC45A4,* were linked with a worse prognosis (Figures 11A,C,G), while expression of *SLC4A5* was not significantly connected with clinical outcome in the Kaplan-Meier survival analysis (Figure 11E). In the GSE21257 cohort, higher *SLC38A5* expression was related with worse overall survival (Figure 11B), while *SLC8A3*, *SLC4A5*, and *SLC45A4* expression was not associated with prognosis.

## Discussion

With the advancement and popularity of high-throughput sequencing technology, discovering genetic abnormalities linked with tumors by large-scale RNA-seq transcriptome sequencing has become a common method in cancer research (Kamps et al., 2017; Hong et al., 2020; Zhao et al., 2020). Some of these transcriptome data were uploaded in opening databases and were re-analyzed by other researchers to discover prognostic indicators and therapeutic targets in a variety of malignancies (Xu et al., 2021b; Deng et al., 2021; Tang et al., 2021). In a previous research, Xiao et al. (2021) mined the expression data of cancer stem cell (CSC)-related genes and the relevant clinical information of osteosarcoma sample from the TCGA and GEO databases, and used them to construct an overall survival prediction signature in osteosarcoma. Liu et al. (2021) used GTEx and TCGA datasets to conduct an integrated study of epigenetic modification-related genes in osteosarcoma, and they developed a powerful epigenetic modification-related prognostic signature that predicted overall survival in osteosarcoma patients. These investigations pave the way for the discovery of prognostic genes in osteosarcoma.

Despite the fact that SLC proteins were clearly crucial to health and illness, they have received far less attention over the past few years (César-Razquin et al., 2015). Many SLC proteins have been identified to be oncogenic or tumor suppressive in malignancies, and aberrant expression of these SLCs might be served as prognosis indicators in various types of tumors (Yan et al., 2015; Wang et al., 2017; Guo et al., 2020). For example, in individuals with colorectal cancer, increased SLC2A3 expression was related with decreased overall survival and disease-free survival (Kim et al., 2019), while increased SLC16A5 expression predicted increased survival in prostate cancer patients (Meng et al., 2021). In osteosarcoma, abnormal expressions of SCLs were associated with increased cell abilities to proliferate, invade, and metastasize. The mitochondrial transporter SLC25A22 is highly increased in osteosarcoma and is associated with a poor prognosis in osteosarcoma patients. SLC25A22 gain and loss of function revealed that it increased cell proliferation, invasion, and metastasis through altering the PTEN signaling pathway (Chen and Wu, 2018). SLC3A2, an integrin-associated protein, was shown to be up-regulated in osteosarcoma samples and cell lines, and its expression was linked to tumor size and stage in osteosarcoma (Zhu et al., 2017). SLC3A2 knockdown decreased cell proliferation by causing cell cycle arrest, which was mediated by PI3K/AKT signaling suppression. Further example includes SLC25A10, which operates as an oncogene in osteosarcoma. In individuals with osteosarcoma, higher expression of SLC25A10 was associated with unfavorable clinicopathological characteristics. Functionally, SLC25A10 positively regulated cell proliferation through regulating the expression of Cyclin E1 (CCNE1), p21 and p27 (Wang et al., 2020a). However, the clinical significance of the SLC family genes in osteosarcoma needs to be further elucidated.

Given the importance of SLCs in the development and progression of osteosarcoma, we undertake a systematic investigation of SLC genes in osteosarcoma using opening databases. Univariate Cox regression analysis was performed to identify prognosis-related SLC genes, which were then utilized to build a prognostic signature in osteosarcoma. This signature enabled patients to be categorized as high-risk or low-risk. Kaplan-Meier survival analysis in all internal and external cohorts revealed that the overall survival of patients in the high-risk group was consistently worse than that of patients in the low-risk group, indicating the promising accuracy and generalizability of the SLC-based signature in predicting the prognosis of osteosarcoma patients. Time-dependent ROC curves confirmed the specificity and accuracy of our SLC-based signature which was superior to some previously reported prognostic signatures in osteosarcoma (Fu et al., 2021; Zhang et al., 2021), since the AUC values of the ROC curves were higher in our research. Furthermore, when patients were divided into different subgroups based on clinical parameters such as gender and age, the prognosis was still poorer in the high-risk group than in the low-risk group in all subgroups, suggesting the signature’s risk stratification utility. Taken together, these findings showed that our SLC-based signature might be a predictive biomarker for patients with osteosarcoma. Furthermore, we created a predictive nomogram based on the risk score and clinicopathological characteristics such as gender and age, which might be useful for clinical decision-making and establishing tailored treatment plans for osteosarcoma patients. To identify possible biological processes and pathways linked with the SLC-based signature, differentially expressed genes between high- and low-risk groups were identified and were subjected to functionally enrichment analysis. The finding revealed that immune-related pathways were significantly enriched. We then used the ESTIMATE algorithm and ssGSEA to investigate the relationship between the signature and the tumor immune microenvironment. We discovered that patients with higher stromal, immune, and ESTIMATE scores had better overall survival, while patients with higher tumor purity had shorter lives than those with lower tumor purity. These findings were consistent with previous researches (Zhang et al., 2020a; He et al., 2021) and suggested that the immunological state of the immune microenvironment was a predictor of overall survival in osteosarcoma patients. Moreover, we discovered that patients in the low-risk group had higher stromal scores, immune scores, ESTIMATE scores, and lower tumor purities. Thus, our four-SLC gene signature was found to be strongly related to the tumor immune microenvironment and may be utilized to predict the efficacy of immune treatment. Furthermore, we discovered that the scores of various immune subpopulations, such as CD8+ T cells, DCs, and TIL, as well as related immune functions, differed considerably between high- and low-risk groups. These findings revealed that the SLC-based signature was strongly related to the immunological status of osteosarcoma and was linked with unique immune cell proportions.

The four SLC genes included *SLC8A3*, *SLC38A5*, *SLC45A4*, and *SLC4A5*. Of the four genes, SLC8A3 expression was higher in the high-risk group, and it was related with a worse outcome, suggesting that it might be oncogenic. SLC8A3, which encodes the Na(+)/Ca(2+) exchanger 3 (NCX3), was shown to be tissue-specific (Gabellini et al., 2002). SLC8A3 mediated the fluxes of Ca(2+) across the membrane and abnormal regulation of *SLC8A3* was associated with distorted Ca(2+)-homeostasis in cells (Khananshvili, 2013). Aberrant expression of *SLC8A3* has been linked to disorders such as arrhythmia, cerebral ischemia, hypertension, heart failure, and diabetes, although its function in tumors has received less attention. Account for the prognostic value of *SLC8A3* in osteosarcoma, further experiments should be conducted to explore its role in osteosarcoma. The expression of *SLC45A4* was higher in low-risk group and higher expression of *SLC45A4* was associated with a better clinical outcome, so *SLC45A4* might be a tumor suppressor gene in osteosarcoma. SLC45A4, a sucrose transporter, is widely expressed in mammalian organs (Bartölke et al., 2014). SLC45A4 was found to be overexpressed in pancreatic ductal adenocarcinoma (PDA) and was strongly linked to poor overall survival in PDA patients. SLC45A4 silencing inhibited cell growth in PDA cells by reducing glucose absorption and ATP generation (Chen et al., 2021). Thus, SLC45A4 may act as both an oncogene and a tumor suppressor in various types of tumors, and its involvement in osteosarcoma has to be investigated further. *SLC38A5* expression was up-regulated in high-risk group than that in low-risk group, and Kaplan-Meier survival analysis in two independent cohorts revealed that higher expression of *SLC38A5* predicted worse overall survival in osteosarcoma. SLC38A5, also known as SNAT5, is an amino acid-dependent Na(+)/H(+) exchanger that is responsible for the import of amino acid and Na(+) and the efflux of H(+) (Kim et al., 2017). The up-regulation of SLC38A5 was evident in breast cancer samples as well as cell lines, and silencing of SLC38A5 attenuated cell proliferation in TNBC cells accompanied by reduced micropinocytosis, suggesting the oncogenic role of SLC38A5 in TNBC (Ramachandran et al., 2021). However, up to now, little is known about the involvement of SLC38A5 in osteosarcoma and other cancers. Our findings suggest that SLC38A5 may act as an oncogene in osteosarcoma, and its role has to be investigated further *in vitro* and *in vivo*.

Our study has some limitations. First, the sample size of osteosarcoma from the public databases is a little small and no other clinicopathological information such as grade and stage could be found. Second, it will be better if the SLC family-based prognosis signature is validated in a real-world cohort of osteosarcoma patients. However, the cases that we have collected are still not enough for external validation. Besides, not all the SLC genes were enrolled in our study and the detailed role of the SLC genes, especially *SLC38A5*, should be further explored.

In conclusion, we identified a novel prognostic signature based on four SLC family genes that can accurately predict overall survival in osteosarcoma patients. Furthermore, the signature is linked to differences in immunological status and immune cell infiltrations in the tumor microenvironment.

## Data Availability

The original contributions presented in the study are included in the article/Supplementary Material, further inquiries can be directed to the corresponding author.
